# Modelling Worldviews as Stable Metabolisms

**DOI:** 10.3390/e24101476

**Published:** 2022-10-17

**Authors:** Tomas Veloz, Pedro Maldonado

**Affiliations:** 1 Centre Leo Apostel, Vrije Universiteit Brussel, Rue de la Strategie 33, 1060 Brussels, Belgium; 2 Fundación para el Desarrollo Interdisciplinario de la Ciencia, la Tecnología y las Artes, Santiago, Chile; 3 Departamento Ciencias Biologicas, Facultad Ciencias de la Vida, Universidad Andres Bello, Santiago 8370146, Chile

**Keywords:** worldviews, emergence, chemical organization theory

## Abstract

The emergence and evolution of worldviews is a complex phenomenon that requires strong and rigorous scientific attention in our hyperconnected world. On the one hand, cognitive theories have proposed reasonable frameworks but have not reached general modeling frameworks where predictions can be tested. On the other hand, machine-learning-based applications perform extremely well at predicting outcomes of worldviews, but they rely on a set of optimized weights in a neural network that does not comply to a well-founded cognitive framework. In this article, we propose a formal approach used to investigate the establishment of and change in worldviews by recalling that the realm of ideas, where opinions, perspectives and worldviews are shaped, resemble, in many ways, a metabolic system. We propose a general modelization of worldviews based on reaction networks, and a specific starting model based on species representing belief attitudes and species representing belief change triggers. These two kinds of species combine and modify their structures through the reactions. We show that chemical organization theory combined with dynamical simulations can illustrate various interesting features of how worldviews emerge, are maintained and change. In particular, worldviews correspond to chemical organizations, meaning closed and self-producing structures, which are generally maintained by feedback loops occurring within the beliefs and triggers in the organization. We also show how, by inducing the external input of belief change triggers, it is possible to change from one worldview to another, in an irreversible way. We illustrate our approach with a simple example reflecting the formation of an opinion and a belief attitude about a theme, and, next, show a more complex scenario containing opinions and belief attitudes about two possible themes.

## 1. Introduction

Over the last two decades, we have witnessed the explosion of communications in media such as social networks and real-time messaging systems. We are currently subjected to a massive and permanent input of facts, opinions and ideas from diverse sources, and we are able to tell to the world what is on our mind, at any time [1]. This situation is completely novel in history, and deserves our deep scientific attention [2].

Nearly ten years before the rise of real-time digital communications, scholars and politicians spoke about a process of globalization, where worldviews will become closer and more compatible due to the new possibilities driven by massive communications, and the world will somehow tend toward a unified form of organization [3,4].

However, over the last decade, we have witnessed various cases of social and political polarization, with a tendency toward social outbreaks (e.g., Arab spring, Gillette jaunes in France, Chilean outbreak) [5]. The latter has been severely influenced by public opinions in the new realm of digital communications, either intentional or spontaneously. Understanding the way that worldviews are shaped and evolve in the new context of permanent information input is a fundamental question for producing a deeper understanding of our collective activities, as well as potential policy-making and technologies to improve our performance as a society.

The modeling of opinions and worldviews is an active field in complex systems, mostly studied under the frameworks of networks and agent-based model systems [6]. These models are well-suited to study the evolution of opinions, and serve in study optimization and control processes [7]. However, they are not well-suited to study the foundational question of how worldviews emerge [8].

Therefore, in this article, we propose a formal approach used to investigate the emergence of worldviews and their evolution, not focusing on the agents but on the dynamics underlying the establishment of beliefs. To achieve this, we recall that the realm of ideas, where opinions, perspectives and worldviews are shaped, resemble, in many ways, a metabolic system. In simple terms, the analogy states that basic ideas, which we can associate to beliefs reached through deduction, external influences or gathered by experience, play the role of biochemical species, and that worldviews play the role of more robust biochemical machinery, such as cells. Indeed, simple ideas (being molecular species in the metabolic side of the analogy) float around, stick with others when there is affinity (bonding), form complex structures (polymers) and, when large enough, become a self-sustaining whole, or worldview (cell), that evolves developing internal mechanisms of preservation and defense against other ideas; however, it is still always possible that a simple small idea (mutant DNA) can enter into a complex structure and, after a while, change it completely (major transition in evolution).

In light of this analogy, the foundational question of the emergence of worldviews is equivalent to the emergence of metabolic systems, i.e., the beginning of life.

Researchers in biochemistry have explained the assemblage of the first forms of life by integrating the thermodynamics of open chemical systems with a theory of self-production and self-maintainance [9]. Namely, molecules combine forming reaction pathways such that the species consumed in each part of the reaction pathway are produced at another part of the reaction pathway [10]. Simple biochemical systems able to operate self-producing pathways in the right environments, so called-autopoietic systems [11], are thus candidates to be the first life forms.

The theory of autopoiesis inspired human sciences to look at the social realm as an autopoietic system [12], where, instead of molecules, we consider communications to be the fundamental entities in interactions, and where people are mere transmitters and replicators of these communications. Thus, this approach claims that social systems are not formed by people and their ideas, but are autopoietic processes of communication.

The formalization of this idea has been explored very little in the literature [13,14]. However, recent results about the emergence of resilience in self-producing systems, and particularly regarding structural features explaining the emergence of goal-directedness in metabolic-like systems [15,16], suggest that the formal analogy between ideas and biochemical realms can give profound insights toward our understanding of the cognitive and social phenomena.

We will introduce a theoretical framework to study the emergence and stability of worldviews, develop examples to illustrate our approach and conclude with a roadmap for incorporating empirical data to our modeling.

## 2. Modeling Social Systems as Metabolisms

The use of autopoietic systems as a conceptual framework to study social systems was proposed by Luhmann [17]. He introduced the notion of communication as the basis of societies’ structuring and ordering [12]. The concept of communication is defined as the flow produced by the exchange of social symbols, such as economical, legal and political, and the communication flow is carried out through a media representing these symbols, such as money, justice and power, respectively. Since, in a general social system, these structures overlap, communications in one social symbol area may affect the others, and eventually produce feedback mechanisms that tend to regulate the behaviour of the communications as a whole. In [14], a reaction network that represents a toy model of a political system based on Luhmann’s concept of communication was introduced. In it, major social aspects, such as decision powers, law enforcement and public needs, are introduced as general social communications, and, by applying chemical organization theory (see Section 3), they derive various forms of potentially stable social organization, resembling either a monarchy, a state-managed system or an community social system. However, the model’s variables are very abstract and difficult to measure, implying that the model has no direct application.

A different approach that aims to complement more traditional social modeling represented evolutionary game theoretical situations using reaction networks [18]. Since evolutionary game theory is a framework that allows us to study problems associated to individual and collective decision making, the reaction network model has the advantage of prescinding from the typical agents used to model these systems, and provides a dynamical description of the evolution of decisions rather than of the agents deciding.

In particular, a strategy in an agent-based model is an algorithm used to perform decisions, whereas, in the reaction network framework, a strategy is a reaction network with a particular response structure to perturbations. Hence, a change in strategy is reflected by the change from one stable reaction network to another.

Although it is possible to reconstruct fundamental results regarding the dilemma of the emergence of cooperation when applying such a framework, and despite various proposals to utilize this framework in more general decision-making situations [19], as a framework used to study sustainability [20] and resilience [21], there have not been sufficient efforts to clarify the representational advantages of reaction networks over traditional networks and agent-based models, possibly because the real-world interesting applications require complex reaction networks of a large size, which are difficult to build and analyze.

Specifically, self-producing structures require a minimum level of complexity to behave in ways that differ from what can be obtained when modeling with traditional networks, especially with regard to acquiring self-producing features. Namely, interactions in a reaction network consume and produce elements, and are not one-to-one but many-to-many, implying that reactions combine, creating pathways that can form loops and reach closure in intricate ways that cannot be built using traditional networks [22]. Moreover, since self-producing structures are non-linear complex dynamical systems, and, in a realistic model, they are permanently subjected to perturbations, it is not trivial to know whether they will remain the same or evolve into something different, and, when they evolve into something different, it is also difficult to predict what other structure they will evolve to.

For this reason, we propose applying chemical organization theory (COT) to study the emergence of worldviews as a form of emergence of goal-directedness [16]. COT allows for the identification of sub-networks of a reaction network that can operate self-producing pathways, skipping non-linear aspects of the dynamics, and thus can operate at a relatively low computational cost. The set of organizations of a reaction network is a reduction in all of the behavioral possibilities of the reaction network to those that can operate as autopoietic systems in the long run [23]. Although there are a number of caveats necessary to build a direct relation between organizations and stable non-linear complex dynamical systems, and we refer to [24,25,26] for detailed studies on these issues, we will show in the next section that COT provides a first approximation to unveil the establishment of self-producing structures in a general way.

## 3. Reaction Networks and Chemical Organizations

A reaction network is defined by a set of species

$M=\{s_{1},\ldots ,s_{n}\}$ and a set of *reactions* $R=\{r_{1},\ldots ,r_{k}\}$. Each reaction $r_{i}$ represents a transformation between collections of species:

(1) $$r_{i}=c_{i1}s_{1}+\cdots +c_{in}s_{n}\to p_{i1}s_{1}+\cdots +p_{ik}s_{n},$$

where $c_{ij}$ and $p_{ij}$ are non-negative integer numbers representing the amount of reactants and products of the species type $s_{j}$, j=1,⋯,n. When $c_{ij}=0$ and $p_{ij}=0$, we have that the species $s_{j}$ does not participate in the reaction at all, if $c_{ij}=0$ and $p_{ij}>0$, we have that $s_{j}$ is produced and not consumed, if $c_{ij}>0$ and $p_{ij}=0$, we have that $s_{j}$ is consumed and not produced and when $c_{ij}>0$ and $p_{ij}>0$, $s_{j}$ is both consumed and produced.

In order to specify the net-production of species by the reactions, we introduce the stoichiometry matrix $S=\left(s_{ij}\right)=p_{ij}-c_{ij}$. S is a matrix with (n×k) entries. Hence, $s_{j}$ is produced by reaction *i* if $s_{ij}>0$, or consumed if $s_{ij}<0$.

In general, the concentrations of the species change over time and are represented by a vector $s\left(t\right)=(s_{1}\left(t\right),\cdots ,s_{n}\left(t\right))$.

Species change their concentration due to the occurrence of reactions. Therefore, we introduce a representation of a reaction pathway by a process vector **v** of dimension *k* (the number of reactions), where the coordinate *i* of **v** represents the number of times that reaction $r_{i}$ is occurring in the pathway. Normally, the process vector depends on the concentration of species, as it is more likely that abundant reactants react more often. Thus, the process vector is a function of the species concentration **v**(**s**(*t*)).

From here, it is possible to obtain an equation for the change in concentration of the species by the application of a reaction pathway v(s(t)):

(2) $$\frac{ds}{dt}=S\cdot v\left(s\left(t\right)\right),$$

As an example, consider the reaction network M={a,b} and R:

$$\begin{array}{cc} r_{1}: & \emptyset \to s_{1}\\ r_{2}: & s_{1}+s_{2}\to 2s_{2} \end{array}$$

We have that $s_{11}=1-0$, $s_{21}=0-0$, $s_{12}=0-1$ and $s_{22}=2-1$. Thus, the stoichiometric matrix is

$$S=\left(\begin{array}{cc} 1 & 0\\ -1 & 1 \end{array}\right)$$

Now, if we apply the processes $v_{1}=\left(2,1\right)$ and $v_{2}=\left(1,2\right)$, we observe that

$$Sv_{1}=\left(\begin{array}{cc} 1 & 0\\ -1 & 1 \end{array}\right)\left(1,2\right)=\left(1-2,0+2\right)=\left(-1,1\right)$$

and

$$Sv_{2}=\left(\begin{array}{cc} 1 & 0\\ -1 & 1 \end{array}\right)\left(2,1\right)=\left(2-1,0+1\right)=\left(1,1\right)$$

Hence, we can see that $v_{1}$ is a process that does not self-produce the system, because species $s_{1}$ is consumed more than produced and $s_{2}$ is produced more than consumed, whereas $v_{2}$ is a process that does self-produce the system. Indeed, $v_{2}$ overproduces the species of the reaction network.

In order to relate the structure of the reaction network based on a subset of species X⊆M and its possible potential to be persistent and dynamically stable, we define $R_{X}\subseteq R$ as the set of all reactions r∈R such that the reactants of the *r* are in *X*. $R_{X}$ is the set of reactions that can be triggered by *X*:

- *X* is *closed* if the products of all reactions in $R_{X}$ are in *X*. This means that now no species can be generated.
- *X* is *self-maintaining* if there exists a process vector v such that S·v≥0 with v[i]>0 for all *i* such that $r_{i}$ is in $R_{X}$. This means that the reaction network that is triggered when species in *X* are present is able to operate in a way where all of its reactions are active, while no species decrease in concentration.

An *organization* is a set *X* that is closed and self-maintaining. Organizations do not produce any novel species (closed) and their species are able to persist in the long-term (self-maintaining). In a reaction network dynamical system (2), it has been proven that fixed points and periodic orbits correspond to organizations [23,25], in the sense that the species with a positive concentration in the long-term dynamics will be organizations, and a slightly more complex notion called distributed organization maps to all stable behaviors in the phase space in dynamics, including spatial-dependent concentrations [26].

COT has been advanced in many fronts, including applications in chemical systems of diverse nature [27,28], computer-science-related applications [29,30] and for modeling ecological systems [15,31,32]. Theoretically, the relation between organizations and dynamical attractors has been extended to more complicated cases, such as limit cycles and heterocyclic orbits [24,25,26], and to cases where the reaction network structure is able to change [22,33].

We will not deepen the more advanced theoretical aspects of COT, but will show how the basic aspects of dynamical systems formed by reaction networks can be applied to model opinion formation in simple cases, as well as how COT allows us to derive a view of worldview landscapes in situations where multiple possible themes and opinions are possible.

Before we introduce specific models, it is very important to formalize the relation between states and the structure of a reaction networks in a dynamical context.

Given a state s(t), we define its ϵ-abstraction Ω(s(t)) as the set of species that have a concentration larger than ϵ. This corresponds to the condition

$$s_{i}\in \Omega \left(s\left(t\right)\right)\;\mathrm{if}\;\mathrm{and}\;\mathrm{only}\;\mathrm{if}\;s\left(t\right)\left[i\right]>\epsilon$$

The reverse notion allows us to obtain dynamical states from abstractions, so we say that s(t) is an *ϵ*-instance of a set of species *X* if and only if, for all species $s_{i}\in X$, we have s(t)[i]>ϵ, and, for all other species, we have s(t)[i]<ϵ.

## 4. A Basic Model of Worldview Fixation and Change

In order to develop a clear and simplified model of a worldview, we will consider a belief system with respect to a unique theme to have an opinion about. For fixing ideas, consider this theme can be, for example, vaccination, and the worldview of the individual will consider such policies as a solution to a problem, or as a problem in itself.

To be more specific, we will consider three possible states *S*, *U* and *D*, describing satisfaction, uncomfortability and discontent with the theme in question, and call these species belief attitudes. We will assume that the total belief attitude is a constant number and thus the three states are zero-sum. Extreme attitudes *S* = 1, *U* = 1 and *D* = 1 represent total satisfaction, uncomfortability and discontent, respectively. In general, the belief attitude is a weighted (convex) combination of these three belief states that evolves over time and can eventually reach stable states, reflecting a stable worldview. Formally, we represent the zero-sum condition over time as S(t)+U(t)+D(t)=1.

In order to represent how belief attitudes can be influenced, we consider two species *s* and *p* representing our interpretation of facts we are informed about of the theme in question. Species *s* means that we interpret the fact as a solution, and *p* as a problem (e.g., vaccination is a solution/problem). Different to belief attitudes, belief change triggers are not zero-sum, and can grow indefinitely.

Therefore, if, for example, we are in a situation at time $t_{1}$ where $S(t_{1})=1$ (and thus $D\left(t_{1}\right)=U\left(t_{1}\right)=0$), and where $p\left(t_{1}\right)\gg s\left(t_{1}\right)$, it means that the worldview at time $t_{1}$ is entirely satisfied with regard to belief attitude, but, simultaneously, it interprets the theme much more as a problem than as a solution. We would expect, then, in this situation, a change in the belief attitude, so, at some posterior time $t_{2}>t_{1}$, we shall have that $S(t_{2})<1$ and thus $U\left(t_{2}\right)+D\left(t_{2}\right)>0$. The species *s*, *p* are thus called belief change triggers. They can be produced from an internal reflection, or by an interaction with the external world (e.g., people and media).

Belief change triggers can establish positive feedback loops with some belief attitudes, implying that such a belief change trigger would grow autocatalytically in the presence of that belief attitude. In our case, we will consider positive feedback loops between *S* and *s*, representing that the belief state of satisfaction reinforces our belief that the state of affairs is more a solution than a problem, and between *D* and *p*, representing that the state of discontent reinforces the belief that the state of affairs is more a problem than a solution.

Additionally, since belief change triggers represent information that we acquire or produce to reinforce or change our belief attitude, this information might be forgotten after some time. Hence, species *s* and *p* tend to disappear after some time in our model. Therefore, the permanence of belief change triggers is either obtained through feedback loops with belief attitudes or by an external input.

We structure these ideas in a set of hypotheses that will help us to build a reaction network model more precisely:

**Hypothesis** **1.** *In the absence of belief change triggers, spontaneous belief attitudes from D to U and from U to S are possible.*

**Hypothesis** **2.** *S and D catalyze the reproduction of s and p, respectively.The reproduction rate is larger for D than for S.*

**Hypothesis** **3.** *s induces a change from U to S and from D to U, whereas p induces the opposite change. Under equal concentration values, these change rates are equal.*

**Hypothesis** **4.** *Belief change triggers decay over time.*

Following the previous assumptions, we define a reaction network

(3) $$\begin{array}{cc} r_{0}: & D\overset{k_{0}=0.1}{\to }U\;,\;(\mathrm{Hyp}.\;1)\\ r_{1}: & U\overset{k_{1}=0.1}{\to }S\;,\;(\mathrm{Hyp}.\;1)\\ r_{2}: & S+s\overset{k_{2}=0.2}{\to }S+2s\;,\;(\mathrm{Hyp}.\;2)\\ r_{3}: & D+p\overset{k_{3}=0.3}{\to }D+2p\;,\;(\mathrm{Hyp}.\;2)\\ r_{4}: & S+p\overset{k_{4}=0.3}{\to }U+p\;,\;(\mathrm{Hyp}.\;3)\\ r_{5}: & U+p\overset{k_{5}=0.3}{\to }D+p\;,\;(\mathrm{Hyp}.\;3)\\ r_{6}: & D+s\overset{k_{6}=0.3}{\to }U+s\;,\;(\mathrm{Hyp}.\;3)\\ r_{7}: & U+s\overset{k_{7}=0.3}{\to }S+s\;,\;(\mathrm{Hyp}.\;3)\\ r_{8}: & 2p\overset{k_{8}=0.15}{\to }p\;,\;(\mathrm{Hyp}.\;4)\\ r_{9}: & 2s\overset{k_{9}=0.15}{\to }s\;,\;(\mathrm{Hyp}.\;4) \end{array}$$

The rates $k_{i}$, i=1,⋯,9 specify the rate constant of the reactions. These choices are consistent with the hypothesis, and serve as a reference to observe the dynamical properties of the system, but do not play a specific role in the results that we will show here.

We will now illustrate some general properties of the network regarding the establishment of worldviews.

Calculations of organizations of the reaction network, as well as the simulations shown in the following sections, are built using a software developed by the authors and is available in github https://github.com/pmaldona/pyRN/ (accessed on 29 September 2022).

**Figure 1:**
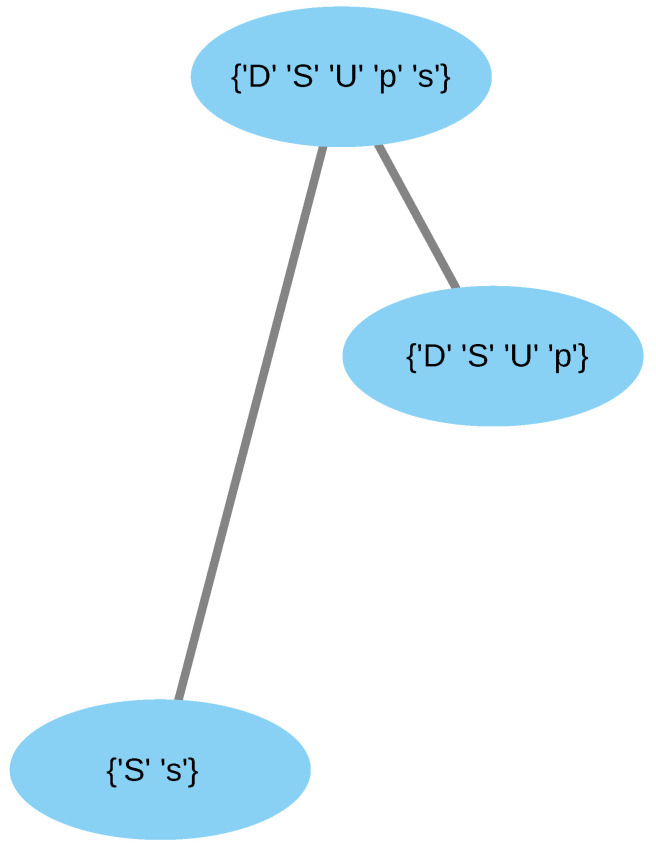
Organizations of the reaction network (3).

As shown in Figure 1, the organizations of this reaction network are $O_{1}=\left\{s,S\right\}$, $O_{2}=\left\{p,S,U,D\right\}$ and $O_{3}=\left\{s,p,S,D,U\right\}$. They form a hierarchy of increasingly more complicated dynamics, where $O_{1}$ represents a situation of complete satisfaction (simple dynamics), and $O_{2}$ represents a situation where, although the three belief attitudes are present, there is only a sense of a problem (less simple dynamics), and, in this sense, $O_{2}$ is the opposite worldview to $O_{1}$. Finally, $O_{3}$ represents a situation where both senses of a solution and problem coexist, implying a wider understanding of the theme (complex dynamics). It is important to remark that different instances of the organizations reflect different ways in which an organization is established.

Therefore, we will simulate the dynamics of the system for identifying the frequency of the different abstractions, being organizations or not, and illustrating the kinds of instances that are most likely to be observed. To achieve this, we built the differential equations for this reaction network, applying the mass action kinetic law [34], a common dynamical law in chemistry and also used regularly in ecology, populations and epidemiology models:

(4) $$\begin{array}{cc} \dot{S} & =k_{1}U-k_{4}Sp+k_{7}Us\\ \dot{U} & =k_{0}D-k_{1}U+k_{4}Sp-k_{5}Up+k_{6}Ds-k_{7}Us\\ \dot{D} & =-k_{0}D+k_{5}Up-k_{6}Ds\\ \dot{p} & =k_{3}Dp-k_{8}p^{2}\\ \dot{s} & =k_{2}Ss-k_{9}s^{2}\\ S\left(0\right)=S_{0},U\left(0\right) & =U_{0},D\left(0\right)=D_{0},p\left(0\right)=p_{0},s\left(0\right)=s_{0} \end{array}$$

### 4.1. No External Influence as Infinite Reflection (with Finite Memory)

In order to get familiarized with the model, let us analyze the simplified situation of starting from certain initial conditions $s\left(0\right)=\left(S_{0},D_{0},1-S_{0}-D_{0},1-p_{0},p_{0}\right)$, which reduce the initial parameters to 3.

By simulating the deterministic evolution of Equation (4), we can identify how different initial states evolve to the different worldviews, represented in our model as organizations.

**Figure 2:**
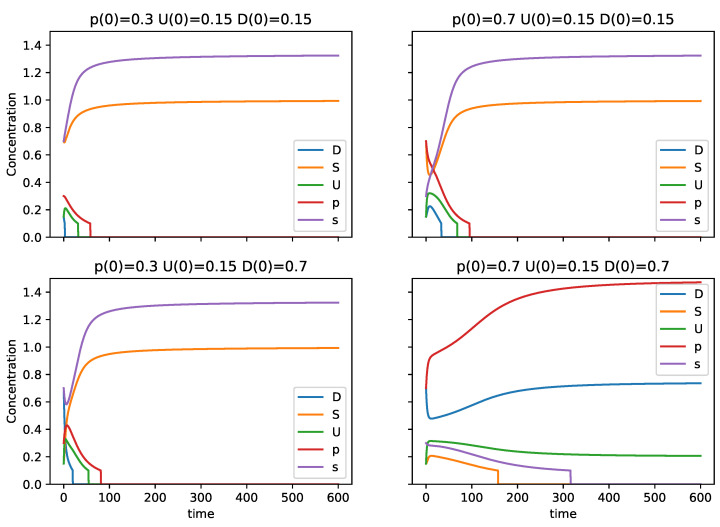
Plots of different initial conditions denoting the tendency for *S* to dominate over *U* and *D*, and for *s* to dominate over *p*, except in circumstances where $p_{0}$ and $D_{0}$ are very large in relation to $s_{0}$ and $S_{0}$.

The plots in Figure 2 show four examples of the dynamics of the system starting from different initial conditions. We see that only when $p_{0}\gg s_{0}$ and $U_{0}\gg S_{0}$ do the dynamics tend to large values for *p* and *D*, and to small values of *S* and *s*, reflecting a tendency toward $O_{2}$. For initial conditions where $S_{0}$ and *s* are larger or comparable to $D_{0}$ and *p*, the dynamics tend to very small values for *D* and *p*, implying a tendency toward $O_{1}$. We see in these cases that *U* is still larger than zero, but, if the dynamics are computed for longer time, *U* will end up decaying as well due to $r_{1}$.

**Figure 3:**
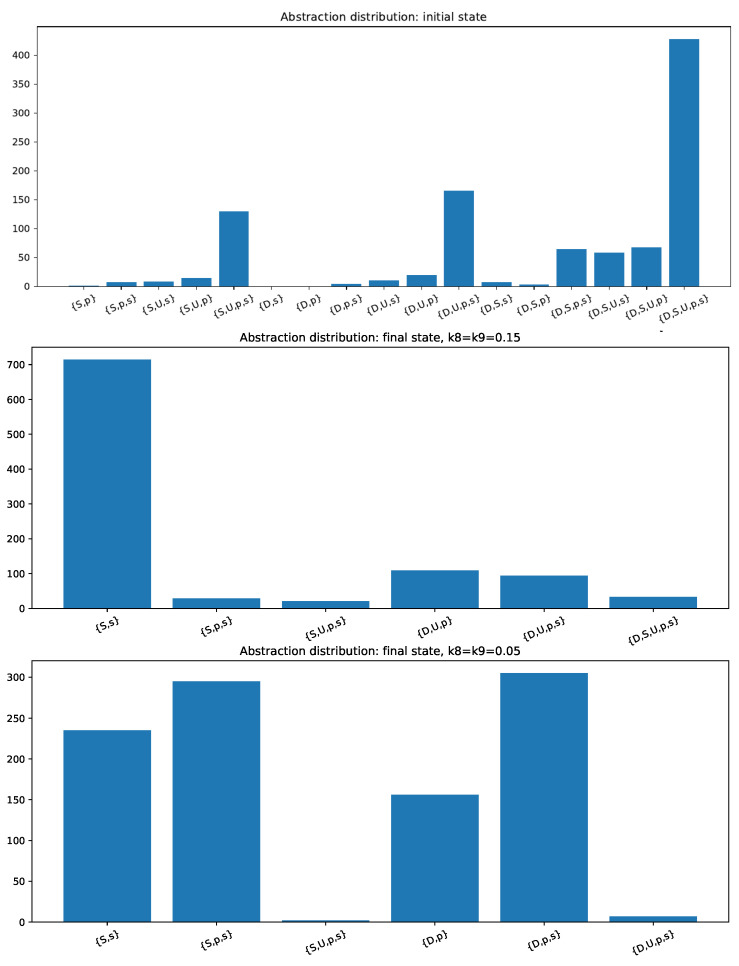
Plots of different initial conditions denoting the tendency for $O_{1}$ dominating over $O_{2}$ when $k_{8}$ and $k_{9}$ are large enough, and noting that such a tendency for dominating is reduced when memory increases, i.e., when $k_{8}=k_{9}=k$ becomes smaller.

For having a better view on the tendency of $O_{1}$ to dominate the dynamics in the space of initial conditions, we calculated the distribution of end states abstractions for *N* = 1000 initial conditions, sampled randomly according to our three-parametric situation. We calculated the abstractions considering ϵ to be the 10% of the total concentration for belief attitudes and for belief change triggers. At the top of Figure 3, we see that the worldview generally begins in a state where all or most species are above the threshold, and it evolves into a state where {S,s} (Figure 3 center) or {p,D,U} (Figure 3 bottom) dominates in frequency. Therefore, we confirm that $O_{1}$ and $O_{2}$ are the most likely end states.

Moreover, we found that the proportion of dominance is strongly dependent on the parameters $k_{8}$ and $k_{9}$, representing the memory of the belief change triggers. Namely, if these parameters become smaller, meaning that the memory of problems and solutions is larger, we observe a tendency to increase the final state where {p,D,U} dominates.

### 4.2. The Emergence of Discontent

Since we see that the parametric situation of the center of Figure 3 ($k_{8}=k_{9}=0.15$) shows the majority of initial conditions that the system evolves towards $O_{1}$, we will show that, by allowing a frequent and strong enough external input of *p*, representing communications that foster the sense of a problem regarding the theme, the system can transit from $O_{1}$ toward $O_{2}$ or $O_{3}$, reflecting the visibilization of problems and discontent.

To this end, we incorporated every $t_{p}$ day an input $\lambda_{p}$ of species *p*. $t_{p}$ and $\lambda_{p}$ are random variables normally distributed with an average of 7 and standard deviation of 1, and homogenously distributed between 0 and $\lambda_{p}=0.25$, respectively.

**Figure 4:**
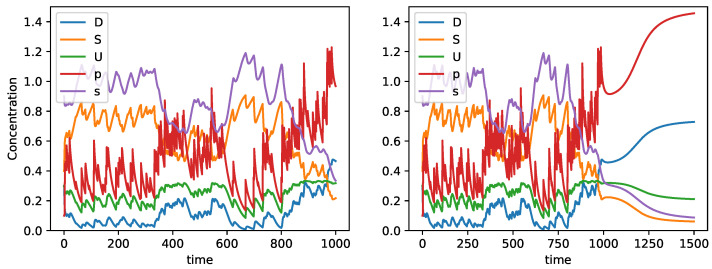
**Left**: Part of the dynamics of the evolution of worldview in situation of satisfaction perturbed with problems; the transition to discontent is not visible. **Right**: Full dynamics; the emergence of discontent occurs between times 1000 and 1500.

In Figure 4, we show a representative example of the dynamics that are obtained in these conditions. Namely, the left plot shows that the input of problems generate oscillations in all variables, implying that the system is kept at $O_{3}$, which we know already from the previous analysis, where it is not a stable dynamical regime in the absence of an input. Over a period of time (which varies between hundreds and a few thousands of iterations), the perturbations generate this oscillatory regime. At a certain point in time, we observe an increase in *D* up to a value where the transition from $O_{3}$ to $O_{2}$ occurs, and thus the values of *S* and *p* become insignificant compared to *D* and *p*. In Figure 4, we observe that such a transition occurs between times 1000 and 1500, but this value is dependent on the random time-series obtained for *p*. In order to see the transition between $O_{3}$ and $O_{2}$ more cleary, we stopped the input of *p* when D(t)>2S(t) in our simulation. In this way, we confirm that $O_{2}$ does not need a permanent input of *p* to stay stable.

The latter analysis demonstrate that worldviews can have radical and non-reversible changes if perturbed in an appropriate way. Further studies regarding the size and frequency of the perturbation, as well as the interplay of inputs of type *s* and *p*, which will reflect communication campaigns dynamics, could be possible.

However, we prefer to focus on the emergence of worldviews in complex scenarios, which is the core subject of this work.

## 5. Modeling a Complex Reaction Network Scenario

In order to exemplify the type of analysis that can potentially be made with these novel methods, we introduce a more complex reaction network, which extends the hypotheses of the reaction network in the previous section, bringing a larger repertoire of possibilities.

**Hypothesis** **5.** *Each belief attitude species is bonded with a belief change trigger about a specific theme, forming a compound belief about a theme.*

**Hypothesis** **6.** *Compound beliefs about different themes are bonded with each other.*

For simplicity, we will consider only two themes, implying that our belief change triggers are $s_{1},s_{2},p_{1},p_{2}$ instead of *s* and *p*. Moreover, Hypotheses 5 and 6 imply that our belief attitudes are compounds formed by belief attitudes and belief change triggers about different themes. For example, $Sp_{1}Us_{2}$ is a species reflecting that, with respect to theme 1 (which can be the legalization of abortion, for example), the belief attitude is satisfaction and is bonded with a perception of a problem (for example, just reading about problems with the legalization of abortion), and with respect to theme 2 (which can be gun policy), there is a sense of uncomfortability bonded with a perception of a solution (for example, reading in the news that the analysis of gun policies shows a decrease in crime rate). Thus, if this compound encounters a species of the kind $p_{1}$ (hearing a story about how bad it is to not improve abortion policies), it will possibly change into $Up_{1}Ss_{2}$ (Hypothesis 3), if it encounters $p_{2}$ (hearing a story about how bad it is to not improve gun policies), it might evolve into $Sp_{1}Up_{2}$ (Hypothesis 3) and, if it does not encounter anything (so not thinking about the themes), it should evolve into a state closer to satisfaction, being either $Sp_{1}Ss_{2}$, or to $Ss_{1}Us_{2}$ (Hypothesis 1).

**Figure 5:**
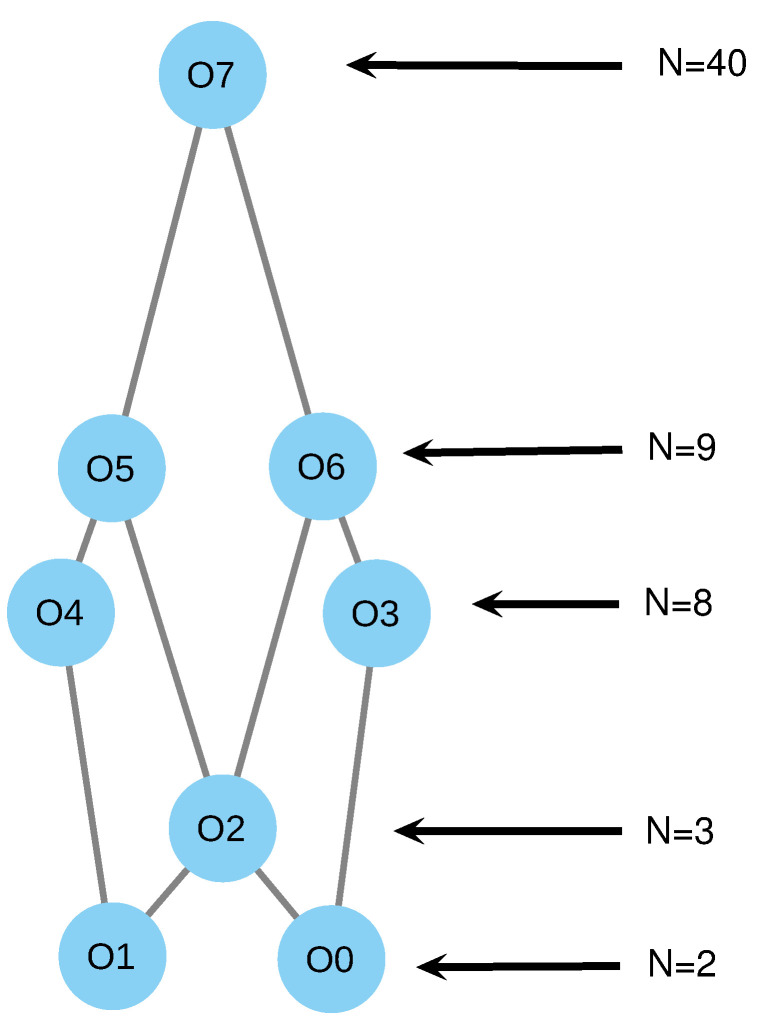
Hasse diagram of the different organizations of the complex model, described in Table 1.

The full list of species and reactions of this reaction network is given in Appendix A, its organizational structure is depicted in Figure 5 and a full description of the organizations is given in Table 1.

First, we notice that the set of organizations is extremely small compared with the total set of subsets of the reaction network. Indeed, since the number of species is 40, the total number of possible abstractions is $2^{40}$.

The simplest organizations $O_{1}$ and $O_{2}$ reflect satisfaction with respect to one theme and no reactivity with respect to the other theme. Indeed, we see that there is a belief change trigger for one solution in each case. $O_{3}$ reflects satisfaction with respect to the two themes. Next, we see that $O_{4}$ and $O_{5}$ incorporate $p_{1}$ and $p_{2}$, respectively, meaning that, with respect to one theme, there is a coexistence of a problem and solution, and, with respect to the other, there is no reactivity. Next, $O_{6}$ and $O_{7}$ incorporate reactivity for $s_{2}$ and $s_{1}$, respectively, implying a sense of a problem and solution in one side, and only a solution in the other. Finally, $O_{7}$ contains all of the species in the set, meaning that the two themes have enough structure for the coexistence of the solution and problematization.

**Figure 6:**
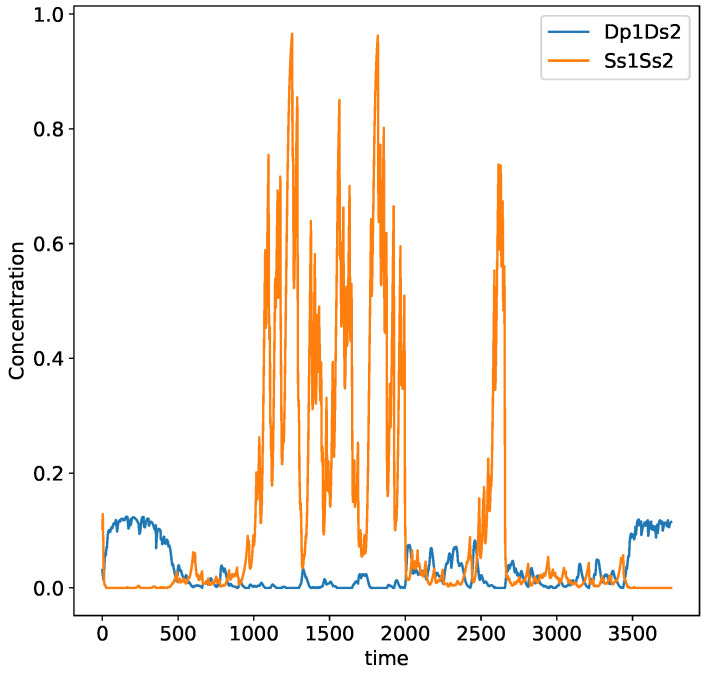
Example of dynamical evolution of species $Ss_{1}Ss_{2}$ and $Dp_{1}Ds_{2}$. Transitions from dominance of satisfaction and discontent are visible.

In order to visualize the dynamics of changes in worldviews in this setting, we developed a similar simulation to the one in Section 4.2, with 1000 perturbations every $t_{p}$ number of days (normal distributed with an average of 7 and standard deviation of 3.5) and with the size of the perturbation homogeneously distributed between 0 and $\lambda_{p}=0.3$, the perturbations including species $p_{1}$ and $p_{2}$ only. In Figure 6, we plot the concentrations of $Ss_{1}Ss_{2}$ and $Dp_{1}Dp_{2}$ to illustrate how the two most extreme belief compounds change over time in the dynamics. We notice that there is a permanent transition, where $Ss_{1}Ss_{2}$ reaches extremely high values and drops radically, whereas $Dp_{1}Dp_{2}$ grows but does not reach values larger than 0.6. The latter means that the other composed species, e.g., $Sp_{1}Us_{2}$ and $U_{p}1Dp_{1}$, also reach significant values over that process (remember that the total belief change attitudes is a constant number). This suggests that the dynamics is very rich in state changes and in what species dominate over time.

**Figure 7:**
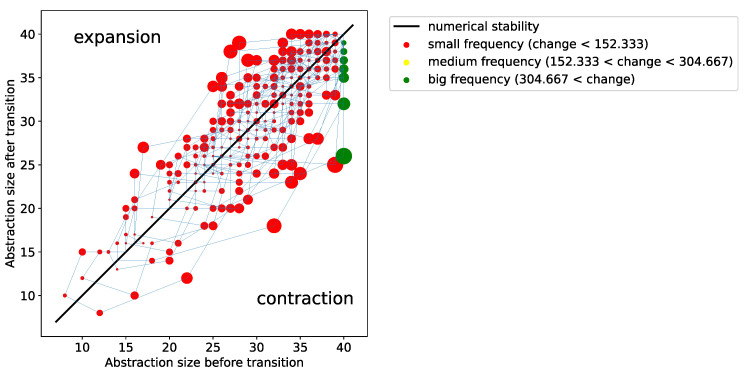
Illustration of the structural evolution of the complex reaction network.

In order to have a better view on what species dominate the dynamics, we developed an illustration in Figure 7 that shows the size of the abstractions reached during the dynamics, and the abstractions are connected by a semitransparent blue line so it is possible to trace the time evolution (the starting abstraction is $O_{3}$ at the bottom left). In this way, we can picture the structural changes that are possible in the dynamics. The color of the point indicates the relative frequency of the abstraction, and the size of the point indicates a measure of change $d(X_{1},X_{2})$ between abstractions $X_{1}$ and $X_{2}$ according to the following function:

$$d\left(X_{1},X_{2}\right)=\frac{|\left(X_{1}\cup X_{2}\right)-\left(X_{1}\cap X_{2}\right)|}{|X_{1}\cup X_{2}|}.$$

The diagonal in the plot indicates that there is no change in size between two successive abstractions, but, still, if we see points of different sizes lying in the diagonal, it means that structural changes do not change the size of the reaction network (e.g., changing from $O_{3}$ to $O_{4}$). Additionally, points above the diagonal imply that the dynamical process increases the size of the active reaction network (dynamical expansion), whereas points below the diagonal imply that the abstraction has reduced its size (dynamical contraction).

The total number of different abstractions visited by the dynamics in the plot is 1208. This is a large number compared with the set of organizations, but still very small compared with the total number of abstractions. The most visited abstraction by far is the full set, as the green points (most frequent) are at the extreme of the X-axis, but, analogous to the previous section, the full set seem unstable and thus leads to several other states, which obviously reflect contractions of the full abstraction. Interestingly, we observe that such contractions are unstable as well, as they reach back to the $O_{7}$.

Therefore, the perturbed dynamics simulated here moves around $O_{7}$, but, different to the simulation found in the previous section, it is not possible to reach a transition where solution species decay, meaning that either $s_{1}$ or $s_{2}$ remain active.

## 6. Conclusions

We have introduced a novel method used to study the formation of worldviews following the analogy between ideas and biochemical species, where worldviews are represented as stable metabolisms. We applied the formalism of reaction networks, and especially COT, to formalize a structural description that is scalable to large and complex reaction networks, where complex themes with multiple possible opinions and belief change repertoires are possible. In our framework, a worldview is an organization, meaning a reaction network that is closed and self-maintaining. We find that belief attitudes ensure their presence by establishing positive feedback loops with belief change triggers. Moreover by applying numerical simulations within the range of the parameters of our hypotheses, we conclude that worldviews where satisfaction is dominant are more likely to be obtained than worldviews where discontent is dominant. However, in the context of a permanent input of information, even though the satisfaction is a robust attractor, it is possible to have irreversible transitions to discontent. These rare, but, afters some time, almost certain transitions represent the phenomena of emergent massive waves of discontent observed today in various parts of the world.

We believe that this approach could be extended further in several directions. First, for the simple case analyzed in Section 4, it would be interesting to study further conditions between the expected time for the transition from $O_{1}$ and $O_{2}$ to happen as a function of the perturbation parameters and the memory, as well as considering other belief change triggers, not just associated to a solution and problem, but also to other sensations, such as how important the theme is (very necessary, unnecessary), or how complex the problem is. Additionally, belief attitudes and belief change triggers can be complemented with other relevant aspects of worldview formations, such as how to decide what actions to carry out, how to build a view on the consequences of such actions, and others.

Referring back to the analysis of the more complex case (Section 5), note that the pairs $O_{4}$ and $O_{5}$ and $O_{6}$ and $O_{7}$ are indeed contradictory perspectives in increasingly more complex ways. The first case sees a problem in one theme and does not have any dynamics in the other, whereas, in the second case, in one theme, it reaches the complexity necessary for problematization, whereas, in the other, it only has satisfaction dynamics. This shows that the emerging worldviews can have an unawareness of themes, or completely opposite views on what is problematic. This indicates that worldviews can not only be opposite, but can also have different kinds of complexities, meaning that they might be compatible or incompatible in some respects (those in which the opposite species of the same type are present), while non-comparable in other respects.

Moreover, if we allow a larger set of belief change triggers in the more complex case, we would indeed provide a way to enrich the descriptive capacity of the model because, in our model, themes 1 and 2 do not interact. Therefore, it would be interesting to incorporate interactions between belief change triggers. For example, if $p_{1}$ fosters the reproduction of $p_{2}$, we will see a tendency toward an increase in $Dp_{2}$ when $Dp_{1}$ is present, whereas this is not necessarily the case for the other way around. The latter paves the way for developing causal and correlational structures driving the formation of worldviews, and thus certain features of a theme could imply other features for other belief change triggers. This would eventually allow for explaining how extremist worldviews, such as anti-vaccination or racist views, tend to explain a variety of social phenomena following very simple precepts, even when they are not properly justified. We believe that such opinion dynamics can be explained by the establishment of positive feedbacks that reinforce their belief attitudes and ensure the permanence of belief change triggers that accommodate the actual worldview (organization).

Regarding a more empirical side for this research, it is necessary to study data regarding opinions, such as polls and social network sentiment analysis data. In this way, it is possible to test this kind of model and contrast it with data. We are still in an early stage of development regarding how to measure complex opinions using natural language processing and other tools [35,36], but we suggest that the qualitative power of COT could be applied to develop novel methods used to measure public opinion.

We believe that the metabolic modeling of opinions and worldviews can be a powerful new scheme for understanding the emergence and dynamics of worldviews.

## Figures and Tables

**Table 1:** List of organizations of complex reaction network. Each organization represents a different worldview. Columns 1–4 represent the organization, species in it, belief change triggers that are reactive in it and its size.

Org.	Species	Triggers	Size
$O_{0}$	$Ss_{1}Ss_{2},s_{1}$	$s_{1}$	2
$O_{1}$	$Ss_{1}Ss_{2},s_{1}$	$s_{2}$	2
$O_{2}$	$Ss_{1},Ss_{2},s_{1},s_{2}$	$s_{1},s_{2}$	3
$O_{3}$	$O_{1}\cup Dp_{1}Ss_{2},Ds_{1}Ss_{2},Sp_{1}Ss_{2},Up_{1}Ss_{2},Us_{1}Ss_{2},p1$	$s_{1},p_{1}$	8
$O_{4}$	$O_{2}\cup Ss_{1}Dp_{2},Ss_{1}Ds_{2},Ss_{1}Sp_{2},Ss_{1}Up_{2},Ss_{1}Us_{2},p_{2}$	$s_{2},p_{2}$	8
$O_{5}$	$O_{3}\cup s_{2}$	$s_{1},p_{1},s_{2}$	9
$O_{6}$	$O_{4}\cup s_{1}$	$s_{2},p_{2},s_{1}$	9
$O_{7}$	M (all species)	$s_{1},p_{1},s_{2},p_{2}$	40

## Data Availability

The software and data required to reproduce the above findings are available to https://github.com/pmaldona/pyRN.

## Appendix A. Complex Reaction Network

### Appendix A.1. Species Set

We built the set of species following all possible combinations that follow Hypotheses 5 and 6 (Section 5). The last four species in the set correspond to the belief change triggers for two themes of the worldview.

$$\begin{array}{cc} M & =\{Dp_{1}Dp_{2},Dp_{1}Ds_{2},Dp_{1}Sp_{2},Dp_{1}Ss_{2},Dp_{1}Up_{2},Dp_{1}Us_{2},\\ & Ds_{1}Dp_{2},Ds_{1}Ds_{2},Ds_{1}Sp_{2},Ds_{1}Ss_{2},Ds_{1}Up_{2},Ds_{1}Us_{2},\\ \; & Sp_{1}Dp_{2},Sp_{1}Ds_{2},Sp_{1}Sp_{2},Sp_{1}Ss_{2},Sp_{1}Up_{2},Sp_{1}Us_{2},\\ \; & Ss_{1}Dp_{2},Ss_{1}Ds_{2},Ss_{1}Sp_{2},Ss_{1}Ss_{2},Ss_{1}Up_{2},Ss_{1}Us_{2},\\ \; & Up_{1}Dp_{2},Up_{1}Ds_{2},Up_{1}Sp_{2},Up_{1}Ss_{2},Up_{1}Up_{2},Up_{1}Us_{2},\\ \; & Us_{1}Dp_{2},Us_{1}Ds_{2},Us_{1}Sp_{2},Us_{1}Ss_{2},Us_{1}Up_{2},Us_{1}Us_{2},p_{1},\\ \; & p_{2},s_{1},s_{2}\} \end{array}$$

### Appendix A.2. Reactions Set

We organized the reactions in groups according to the hypothesis that they are a consequence of:

- Reactions $\left\{r_{0},\cdots ,r_{59}\right\}$ correspond to reactions according to Hypothesis 1
- Reactions $\left\{r_{60},\cdots ,r_{119}\right\}$ correspond to reactions according to Hypothesis 2
- Reactions $\left\{r_{120},\cdots ,r_{143}\right\}$ correspond to reactions according to Hypothesis 3
- Reactions $\left\{r_{144},\cdots ,r_{147}\right\}$ correspond to reactions according to Hypothesis 4.

$$\begin{array}{cccccccc} r_{0}: & Ds_{1}Ds_{2}\overset{k_{0}=0.1}{\to }Us_{1}Ds_{2} & \; & r_{54}: & Ss_{1}Dp_{2}\overset{k_{0}=0.1}{\to }Ss_{1}Ds_{2} & \; & r_{108}: & Ss_{1}Sp_{2}+p_{2}\overset{k_{0}=0.3}{\to }Ss_{1}Up_{2}+p_{2}\\ r_{1}: & Ds_{1}Dp_{2}\overset{k_{0}=0.1}{\to }Us_{1}Dp_{2} & \; & r_{55}: & Sp_{1}Dp_{2}\overset{k_{0}=0.1}{\to }Sp_{1}Ds_{2} & \; & r_{109}: & Sp_{1}Sp_{2}+p_{2}\overset{k_{0}=0.3}{\to }Sp_{1}Up_{2}+p_{2}\\ r_{2}: & Ds_{1}Us_{2}\overset{k_{0}=0.1}{\to }Us_{1}Us_{2} & \; & r_{56}: & Us_{1}Dp_{2}\overset{k_{0}=0.1}{\to }Us_{1}Ds_{2} & \; & r_{110}: & Us_{1}Sp_{2}+p_{2}\overset{k_{0}=0.3}{\to }Us_{1}Up_{2}+p_{2}\\ r_{3}: & Ds_{1}Up_{2}\overset{k_{0}=0.1}{\to }Us_{1}Up_{2} & \; & r_{57}: & Up_{1}Dp_{2}\overset{k_{0}=0.1}{\to }Up_{1}Ds_{2} & \; & r_{111}: & Up_{1}Sp_{2}+p_{2}\overset{k_{0}=0.3}{\to }Up_{1}Up_{2}+p_{2}\\ r_{4}: & Ds_{1}Ss_{2}\overset{k_{0}=0.1}{\to }Us_{1}Ss_{2} & \; & r_{58}: & Ds_{1}Dp_{2}\overset{k_{0}=0.1}{\to }Ds_{1}Ds_{2} & \; & r_{112}: & Ds_{1}Sp_{2}+p_{2}\overset{k_{0}=0.3}{\to }Ds_{1}Up_{2}+p_{2}\\ r_{5}: & Ds_{1}Sp_{2}\overset{k_{0}=0.1}{\to }Us_{1}Sp_{2} & \; & r_{59}: & Dp_{1}Dp_{2}\overset{k_{0}=0.1}{\to }Dp_{1}Ds_{2} & \; & r_{113}: & Dp_{1}Sp_{2}+p_{2}\overset{k_{0}=0.3}{\to }Dp_{1}Up_{2}+p_{2}\\ r_{6}: & Us_{1}Ds_{2}\overset{k_{0}=0.1}{\to }Ss_{1}Ds_{2} & \; & r_{60}: & Ss_{1}Ss_{2}+p_{1}\overset{k_{0}=0.3}{\to }Sp_{1}Ss_{2}+s_{1} & \; & r_{114}: & Ss_{1}Up_{2}+p_{2}\overset{k_{0}=0.3}{\to }Ss_{1}Dp_{2}+p_{2}\\ r_{7}: & Us_{1}Dp_{2}\overset{k_{0}=0.1}{\to }Ss_{1}Dp_{2} & \; & r_{61}: & Ss_{1}Sp_{2}+p_{1}\overset{k_{0}=0.3}{\to }Sp_{1}Sp_{2}+s_{1} & \; & r_{115}: & Sp_{1}Up_{2}+p_{2}\overset{k_{0}=0.3}{\to }Sp_{1}Dp_{2}+p_{2}\\ r_{8}: & Us_{1}Us_{2}\overset{k_{0}=0.1}{\to }Ss_{1}Us_{2} & \; & r_{62}: & Ss_{1}Us_{2}+p_{1}\overset{k_{0}=0.3}{\to }Sp_{1}Us_{2}+s_{1} & \; & r_{116}: & Us_{1}Up_{2}+p_{2}\overset{k_{0}=0.3}{\to }Us_{1}Dp_{2}+p_{2}\\ r_{9}: & Us_{1}Up_{2}\overset{k_{0}=0.1}{\to }Ss_{1}Up_{2} & \; & r_{63}: & Ss_{1}Up_{2}+p_{1}\overset{k_{0}=0.3}{\to }Sp_{1}Up_{2}+s_{1} & \; & r_{117}: & Up_{1}Up_{2}+p_{2}\overset{k_{0}=0.3}{\to }Up_{1}Dp_{2}+p_{2}\\ r_{10}: & Us_{1}Ss_{2}\overset{k_{0}=0.1}{\to }Ss_{1}Ss_{2} & \; & r_{64}: & Ss_{1}Ds_{2}+p_{1}\overset{k_{0}=0.3}{\to }Sp_{1}Ds_{2}+s_{1} & \; & r_{118}: & Ds_{1}Up_{2}+p_{2}\overset{k_{0}=0.3}{\to }Ds_{1}Dp_{2}+p_{2}\\ r_{11}: & Us_{1}Sp_{2}\overset{k_{0}=0.1}{\to }Ss_{1}Sp_{2} & \; & r_{65}: & Ss_{1}Dp_{2}+p_{1}\overset{k_{0}=0.3}{\to }Sp_{1}Dp_{2}+s_{1} & \; & r_{119}: & Dp_{1}Up_{2}+p_{2}\overset{k_{0}=0.3}{\to }Dp_{1}Dp_{2}+p_{2}\\ r_{12}: & Ds_{1}Ds_{2}\overset{k_{0}=0.1}{\to }Ds_{1}Us_{2} & \; & r_{66}: & Us_{1}Ss_{2}+p_{1}\overset{k_{0}=0.3}{\to }Up_{1}Ss_{2}+s_{1} & \; & r_{120}: & Ss_{1}Ss_{2}+s_{1}\overset{k_{0}=0.2}{\to }Ss_{1}Ss_{2}+2s_{1}\\ r_{13}: & Dp_{1}Ds_{2}\overset{k_{0}=0.1}{\to }Dp_{1}Us_{2} & \; & r_{67}: & Us_{1}Sp_{2}+p_{1}\overset{k_{0}=0.3}{\to }Up_{1}Sp_{2}+s_{1} & \; & r_{121}: & Ss_{1}Sp_{2}+s_{1}\overset{k_{0}=0.2}{\to }Ss_{1}Sp_{2}+2s_{1}\\ r_{14}: & Us_{1}Ds_{2}\overset{k_{0}=0.1}{\to }Us_{1}Us_{2} & \; & r_{68}: & Us_{1}Us_{2}+p_{1}\overset{k_{0}=0.3}{\to }Up_{1}Us_{2}+s_{1} & \; & r_{122}: & Ss_{1}Us_{2}+s_{1}\overset{k_{0}=0.2}{\to }Ss_{1}Us_{2}+2s_{1}\\ r_{15}: & Up_{1}Ds_{2}\overset{k_{0}=0.1}{\to }Up_{1}Us_{2} & \; & r_{69}: & Us_{1}Up_{2}+p_{1}\overset{k_{0}=0.3}{\to }Up_{1}Up_{2}+s_{1} & \; & r_{123}: & Ss_{1}Up_{2}+s_{1}\overset{k_{0}=0.2}{\to }Ss_{1}Up_{2}+2s_{1}\\ r_{16}: & Ss_{1}Ds_{2}\overset{k_{0}=0.1}{\to }Ss_{1}Us_{2} & \; & r_{70}: & Us_{1}Ds_{2}+p_{1}\overset{k_{0}=0.3}{\to }Up_{1}Ds_{2}+s_{1} & \; & r_{124}: & Ss_{1}Ds_{2}+s_{1}\overset{k_{0}=0.2}{\to }Ss_{1}Ds_{2}+2s_{1}\\ r_{17}: & Sp_{1}Ds_{2}\overset{k_{0}=0.1}{\to }Sp_{1}Us_{2} & \; & r_{71}: & Us_{1}Dp_{2}+p_{1}\overset{k_{0}=0.3}{\to }Up_{1}Dp_{2}+s_{1} & \; & r_{125}: & Ss_{1}Dp_{2}+s_{1}\overset{k_{0}=0.2}{\to }Ss_{1}Dp_{2}+2s_{1}\\ r_{18}: & Ds_{1}Us_{2}\overset{k_{0}=0.1}{\to }Ds_{1}Ss_{2} & \; & r_{72}: & Ds_{1}Ss_{2}+p_{1}\overset{k_{0}=0.3}{\to }Dp_{1}Ss_{2}+s_{1} & \; & r_{126}: & Dp_{1}Ss_{2}+p_{1}\overset{k_{0}=0.3}{\to }Dp_{1}Ss_{2}+2p_{1}\\ r_{19}: & Dp_{1}Us_{2}\overset{k_{0}=0.1}{\to }Dp_{1}Ss_{2} & \; & r_{73}: & Ds_{1}Sp_{2}+p_{1}\overset{k_{0}=0.3}{\to }Dp_{1}Sp_{2}+s_{1} & \; & r_{127}: & Dp_{1}Sp_{2}+p_{1}\overset{k_{0}=0.3}{\to }Dp_{1}Sp_{2}+2p_{1}\\ r_{20}: & Us_{1}Us_{2}\overset{k_{0}=0.1}{\to }Us_{1}Ss_{2} & \; & r_{74}: & Ds_{1}Us_{2}+p_{1}\overset{k_{0}=0.3}{\to }Dp_{1}Us_{2}+s_{1} & \; & r_{128}: & Dp_{1}Us_{2}+p_{1}\overset{k_{0}=0.3}{\to }Dp_{1}Us_{2}+2p_{1}\\ r_{21}: & Up_{1}Us_{2}\overset{k_{0}=0.1}{\to }Up_{1}Ss_{2} & \; & r_{75}: & Ds_{1}Up_{2}+p_{1}\overset{k_{0}=0.3}{\to }Dp_{1}Up_{2}+s_{1} & \; & r_{129}: & Dp_{1}Up_{2}+p_{1}\overset{k_{0}=0.3}{\to }Dp_{1}Up_{2}+2p_{1}\\ r_{22}: & Ss_{1}Us_{2}\overset{k_{0}=0.1}{\to }Ss_{1}Ss_{2} & \; & r_{76}: & Ds_{1}Ds_{2}+p_{1}\overset{k_{0}=0.3}{\to }Dp_{1}Ds_{2}+s_{1} & \; & r_{130}: & Dp_{1}Ds_{2}+p_{1}\overset{k_{0}=0.3}{\to }Dp_{1}Ds_{2}+2p_{1}\\ r_{23}: & Sp_{1}Us_{2}\overset{k_{0}=0.1}{\to }Sp_{1}Ss_{2} & \; & r_{77}: & Ds_{1}Dp_{2}+p_{1}\overset{k_{0}=0.3}{\to }Dp_{1}Dp_{2}+s_{1} & \; & r_{131}: & Dp_{1}Dp_{2}+p_{1}\overset{k_{0}=0.3}{\to }Dp_{1}Dp_{2}+2p_{1}\\ r_{24}: & Sp_{1}Ss_{2}\overset{k_{0}=0.1}{\to }Ss_{1}Ss_{2} & \; & r_{78}: & Ss_{1}Ss_{2}+p_{2}\overset{k_{0}=0.3}{\to }Ss_{1}Sp_{2}+s_{2} & \; & r_{132}: & Ss_{1}Ss_{2}+s_{2}\overset{k_{0}=0.2}{\to }Ss_{1}Ss_{2}+2s_{2}\\ r_{25}: & Sp_{1}Sp_{2}\overset{k_{0}=0.1}{\to }Ss_{1}Sp_{2} & \; & r_{79}: & Sp_{1}Ss_{2}+p_{2}\overset{k_{0}=0.3}{\to }Sp_{1}Sp_{2}+s_{2} & \; & r_{133}: & Sp_{1}Ss_{2}+s_{2}\overset{k_{0}=0.2}{\to }Sp_{1}Ss_{2}+2s_{2}\\ r_{26}: & Sp_{1}Us_{2}\overset{k_{0}=0.1}{\to }Ss_{1}Us_{2} & \; & r_{80}: & Us_{1}Ss_{2}+p_{2}\overset{k_{0}=0.3}{\to }Us_{1}Sp_{2}+s_{2} & \; & r_{134}: & Us_{1}Ss_{2}+s_{2}\overset{k_{0}=0.2}{\to }Us_{1}Ss_{2}+2s_{2}\\ r_{27}: & Sp_{1}Up_{2}\overset{k_{0}=0.1}{\to }Ss_{1}Up_{2} & \; & r_{81}: & Up_{1}Ss_{2}+p_{2}\overset{k_{0}=0.3}{\to }Up_{1}Sp_{2}+s_{2} & \; & r_{135}: & Up_{1}Ss_{2}+s_{2}\overset{k_{0}=0.2}{\to }Up_{1}Ss_{2}+2s_{2}\\ r_{28}: & Sp_{1}Ds_{2}\overset{k_{0}=0.1}{\to }Ss_{1}Ds_{2} & \; & r_{82}: & Ds_{1}Ss_{2}+p_{2}\overset{k_{0}=0.3}{\to }Ds_{1}Sp_{2}+s_{2} & \; & r_{136}: & Ds_{1}Ss_{2}+s_{2}\overset{k_{0}=0.2}{\to }Ds_{1}Ss_{2}+2s_{2}\\ r_{29}: & Sp_{1}Dp_{2}\overset{k_{0}=0.1}{\to }Ss_{1}Dp_{2} & \; & r_{83}: & Dp_{1}Ss_{2}+p_{2}\overset{k_{0}=0.3}{\to }Dp_{1}Sp_{2}+s_{2} & \; & r_{137}: & Dp_{1}Ss_{2}+s_{2}\overset{k_{0}=0.2}{\to }Dp_{1}Ss_{2}+2s_{2}\\ r_{30}: & Up_{1}Ss_{2}\overset{k_{0}=0.1}{\to }Us_{1}Ss_{2} & \; & r_{84}: & Ss_{1}Us_{2}+p_{2}\overset{k_{0}=0.3}{\to }Ss_{1}Up_{2}+s_{2} & \; & r_{138}: & Ss_{1}Dp_{2}+p_{2}\overset{k_{0}=0.3}{\to }Ss_{1}Dp_{2}+2p_{2}\\ r_{31}: & Up_{1}Sp_{2}\overset{k_{0}=0.1}{\to }Us_{1}Sp_{2} & \; & r_{85}: & Sp_{1}Us_{2}+p_{2}\overset{k_{0}=0.3}{\to }Sp_{1}Up_{2}+s_{2} & \; & r_{139}: & Sp_{1}Dp_{2}+p_{2}\overset{k_{0}=0.3}{\to }Sp_{1}Dp_{2}+2p_{2}\\ r_{32}: & Up_{1}Us_{2}\overset{k_{0}=0.1}{\to }Us_{1}Us_{2} & \; & r_{86}: & Us_{1}Us_{2}+p_{2}\overset{k_{0}=0.3}{\to }Us_{1}Up_{2}+s_{2} & \; & r_{140}: & Us_{1}Dp_{2}+p_{2}\overset{k_{0}=0.3}{\to }Us_{1}Dp_{2}+2p_{2}\\ r_{33}: & Up_{1}Up_{2}\overset{k_{0}=0.1}{\to }Us_{1}Up_{2} & \; & r_{87}: & Up_{1}Us_{2}+p_{2}\overset{k_{0}=0.3}{\to }Up_{1}Up_{2}+s_{2} & \; & r_{141}: & Up_{1}Dp_{2}+p_{2}\overset{k_{0}=0.3}{\to }Up_{1}Dp_{2}+2p_{2}\\ r_{34}: & Up_{1}Ds_{2}\overset{k_{0}=0.1}{\to }Us_{1}Ds_{2} & \; & r_{88}: & Ds_{1}Us_{2}+p_{2}\overset{k_{0}=0.3}{\to }Ds_{1}Up_{2}+s_{2} & \; & r_{142}: & Ds_{1}Dp_{2}+p_{2}\overset{k_{0}=0.3}{\to }Ds_{1}Dp_{2}+2p_{2}\\ r_{35}: & Up_{1}Dp_{2}\overset{k_{0}=0.1}{\to }Us_{1}Dp_{2} & \; & r_{89}: & Dp_{1}Us_{2}+p_{2}\overset{k_{0}=0.3}{\to }Dp_{1}Up_{2}+s_{2} & \; & r_{143}: & Dp_{1}Dp_{2}+p_{2}\overset{k_{0}=0.3}{\to }Dp_{1}Dp_{2}+2p_{2}\\ r_{36}: & Dp_{1}Ss_{2}\overset{k_{0}=0.1}{\to }Ds_{1}Ss_{2} & \; & r_{90}: & Ss_{1}Ds_{2}+p_{2}\overset{k_{0}=0.3}{\to }Ss_{1}Dp_{2}+s_{2} & \; & r_{144}: & 2s_{1}\overset{k_{0}=0.15}{\to }s_{1}\\ r_{37}: & Dp_{1}Sp_{2}\overset{k_{0}=0.1}{\to }Ds_{1}Sp_{2} & \; & r_{91}: & Sp_{1}Ds_{2}+p_{2}\overset{k_{0}=0.3}{\to }Sp_{1}Dp_{2}+s_{2} & \; & r_{145}: & 2s_{2}\overset{k_{0}=0.15}{\to }s_{2}\\ r_{38}: & Dp_{1}Us_{2}\overset{k_{0}=0.1}{\to }Ds_{1}Us_{2} & \; & r_{92}: & Us_{1}Ds_{2}+p_{2}\overset{k_{0}=0.3}{\to }Us_{1}Dp_{2}+s_{2} & \; & r_{146}: & 2p_{1}\overset{k_{0}=0.15}{\to }p_{1}\\ r_{39}: & Dp_{1}Up_{2}\overset{k_{0}=0.1}{\to }Ds_{1}Up_{2} & \; & r_{93}: & Up_{1}Ds_{2}+p_{2}\overset{k_{0}=0.3}{\to }Up_{1}Dp_{2}+s_{2} & \; & r_{147}: & 2p_{2}\overset{k_{0}=0.15}{\to }p_{2}\\ r_{40}: & Dp_{1}Ds_{2}\overset{k_{0}=0.1}{\to }Ds_{1}Ds_{2} & \; & r_{94}: & Ds_{1}Ds_{2}+p_{2}\overset{k_{0}=0.3}{\to }Ds_{1}Dp_{2}+s_{2} & \; & \; & \\ r_{41}: & Dp_{1}Dp_{2}\overset{k_{0}=0.1}{\to }Ds_{1}Dp_{2} & \; & r_{95}: & Dp_{1}Ds_{2}+p_{2}\overset{k_{0}=0.3}{\to }Dp_{1}Dp_{2}+s_{2} & \; & \; & \\ r_{42}: & Ss_{1}Sp_{2}\overset{k_{0}=0.1}{\to }Ss_{1}Ss_{2} & \; & r_{96}: & Sp_{1}Ss_{2}+p_{1}\overset{k_{0}=0.3}{\to }Up_{1}Ss_{2}+p_{1} & \; & \; & \\ r_{43}: & Sp_{1}Sp_{2}\overset{k_{0}=0.1}{\to }Sp_{1}Ss_{2} & \; & r_{97}: & Sp_{1}Sp_{2}+p_{1}\overset{k_{0}=0.3}{\to }Up_{1}Sp_{2}+p_{1} & \; & \; & \\ r_{44}: & Us_{1}Sp_{2}\overset{k_{0}=0.1}{\to }Us_{1}Ss_{2} & \; & r_{98}: & Sp_{1}Us_{2}+p_{1}\overset{k_{0}=0.3}{\to }Up_{1}Us_{2}+p_{1} & \; & \; & \\ r_{45}: & Up_{1}Sp_{2}\overset{k_{0}=0.1}{\to }Up_{1}Ss_{2} & \; & r_{99}: & Sp_{1}Up_{2}+p_{1}\overset{k_{0}=0.3}{\to }Up_{1}Up_{2}+p_{1} & \; & \; & \\ r_{46}: & Ds_{1}Sp_{2}\overset{k_{0}=0.1}{\to }Ds_{1}Ss_{2} & \; & r_{100}: & Sp_{1}Ds_{2}+p_{1}\overset{k_{0}=0.3}{\to }Up_{1}Ds_{2}+p_{1} & \; & \; & \\ r_{47}: & Dp_{1}Sp_{2}\overset{k_{0}=0.1}{\to }Dp_{1}Ss_{2} & \; & r_{101}: & Sp_{1}Dp_{2}+p_{1}\overset{k_{0}=0.3}{\to }Up_{1}Dp_{2}+p_{1} & \; & \; & \\ r_{48}: & Ss_{1}Up_{2}\overset{k_{0}=0.1}{\to }Ss_{1}Us_{2} & \; & r_{102}: & Up_{1}Ss_{2}+p_{1}\overset{k_{0}=0.3}{\to }Dp_{1}Ss_{2}+p_{1} & \; & \; & \\ r_{49}: & Sp_{1}Up_{2}\overset{k_{0}=0.1}{\to }Sp_{1}Us_{2} & \; & r_{103}: & Up_{1}Sp_{2}+p_{1}\overset{k_{0}=0.3}{\to }Dp_{1}Sp_{2}+p_{1} & \; & \; & \\ r_{50}: & Us_{1}Up_{2}\overset{k_{0}=0.1}{\to }Us_{1}Us_{2} & \; & r_{104}: & Up_{1}Us_{2}+p_{1}\overset{k_{0}=0.3}{\to }Dp_{1}Us_{2}+p_{1} & \; & \; & \\ r_{51}: & Up_{1}Up_{2}\overset{k_{0}=0.1}{\to }Up_{1}Us_{2} & \; & r_{105}: & Up_{1}Up_{2}+p_{1}\overset{k_{0}=0.3}{\to }Dp_{1}Up_{2}+p_{1} & \; & \; & \\ r_{52}: & Ds_{1}Up_{2}\overset{k_{0}=0.1}{\to }Ds_{1}Us_{2} & \; & r_{106}: & Up_{1}Ds_{2}+p_{1}\overset{k_{0}=0.3}{\to }Dp_{1}Ds_{2}+p_{1} & \; & \; & \\ r_{53}: & Dp_{1}Up_{2}\overset{k_{0}=0.1}{\to }Dp_{1}Us_{2} & \; & r_{107}: & Up_{1}Dp_{2}+p_{1}\overset{k_{0}=0.3}{\to }Dp_{1}Dp_{2}+p_{1} & \; & \; & \end{array}$$
